# Aberrant expression of RAB1A in human tongue cancer

**DOI:** 10.1038/sj.bjc.6602594

**Published:** 2005-05-03

**Authors:** K Shimada, K Uzawa, M Kato, Y Endo, M Shiiba, H Bukawa, H Yokoe, N Seki, H Tanzawa

**Affiliations:** 1 Department of Clinical Molecular Biology, Graduate School of Medicine, Chiba University, 1-8-1 Inohana, Chuo-ku, Chiba 260-8670, Japan; 2 Department of Functional Genomics, Graduate School of Medicine, Chiba University, 1-8-1 Inohana, Chuo-ku, Chiba 260-8670, Japan; 3 Division of Oral Surgery, Chiba University Hospital, 1-8-1 Inohana, Chuo-ku, Chiba 260-8670, Japan; 4 Center of Excellence (COE) Program in the 21st Century, Graduate School of Medicine, Chiba University, 1-8-1 Inohana, Chuo-ku, Chiba 260-8670, Japan

**Keywords:** tongue squamous cell carcinoma, in-house cDNA microarray, gene expression profiling, *Rab1a* gene

## Abstract

This study was designed to identify specific gene expression changes in tongue squamous cell carcinomas (TSCCs) compared with normal tissues using in-house cDNA microarray that comprised of 2304 full-length cDNAs from a cDNA library prepared from normal oral tissues, primary oral cancers, and oral cancer cell lines. The genes identified by our microarray system were further analysed at the mRNA or protein expression level in a series of clinical samples by real-time quantitative reverse transcriptase–polymerase chain reaction (qRT–PCR) analysis and imuunohositochemistry. The microarray analysis identified a total of 16 genes that were significantly upregulated in common among four TSCC specimens. Consistent with the results of the microarray, increased mRNA levels of selected genes with known molecular functions were found in the four TSCCs. Among genes identified, *Rab1a*, a member of the *Ras* oncogene family, was further analysed for its protein expression in 54 TSCCs and 13 premalignant lesions. We found a high prevalence of Rab1A-overexpression not only in TSCCs (98%) but also in premalignant lesions (93%). Thus, our results suggest that rapid characterisation of the target gene(s) for TSCCs can be accomplished using our in-house cDNA microarray analysis combined with the qRT–PCR and immunohistochemistry, and that the Rab1A is a potential biomarker of tongue carcinogenesis.

Squamous cell carcinoma (SCC) is by far the most common malignant neoplasm of the oral cavity, representing approximately 90% of all oral cancers. Although it occurs at various oral regions, the tongue is one of the most frequent sites (Boyle *et al*, 1992; Perkin *et al*, 1993). A number of aetiologic factors have been implicated in the development of oral SCCs, such as the use of tobacco, alcohol, or the presence of incompatible prosthetic materials (Mashberg *et al*, 1993; Macfarlane *et al*, 1995). However, some patients without those risk factors actually develop tumours of the tongue, and there are individual variations in the progression or differentiation of the cancer, which suggests that host susceptibility may play a role. In this context, molecular alterations in a number of oncogenes and tumour suppressor genes associated with the development of tongue SCC (TSCC) could be important clues for addressing these problems (Fearon and Vogelstein, 1990; Marshall, 1991).

Recent reports have documented alterations of a few oncogenes and tumour suppressor genes in oral cancer including TSCC (Scully *et al*, 2000), but the molecular and genetic basis of tongue carcinogenesis still remains largely unknown and global gene expression has not been clarified. In recent years, the cDNA microarray system has facilitated the simultaneous investigation of a large set of genes or gene clusters, allowing analysis of even complex processes in a short time (Schena *et al*, 1995; DeRisi *et al*, 1996). We recently developed an in-house cDNA microarray derived from our oligo-capped human oral cancer cDNA library (Moriya *et al*, 2003). This microarray system successfully identified genes that are differentially expressed in common in oral SCC-derived cell lines compared with those in normal oral tissues, indicating that this system may be a powerful prioritisation tool in the search for disease significance.

In the present study, we attempted to identify target gene(s) for TSCC using our cDNA microarray. In addition, expression states of the mRNA and protein of candidate genes were evaluated further by real-time quantitative reverse transcriptase–polymerase chain reaction (qRT–PCR) analysis, or both and semiquantitative immunohistochemistry in a large series of tumours of the tongue and premalignant lesions of the tongue (tongue leukoplakias: TLPs).

## MATERIALS AND METHODS

### Tissue samples

Tumours of the tongue or premalignant lesions of the tongue (histologically diagnosed as TLPs), with patient-matched normal epithelium where available, were obtained at the time of surgical resection at Chiba University Hospital after the patient’s informed consent was obtained under a protocol reviewed and approved by the institutional review board of Chiba University. The resected tissues were divided into two parts, one of which was frozen immediately after careful removal of the surrounding normal tissues and stored at −80°C until RNA isolation; the second part was fixed in 10% buffered formaldehyde solution for pathologic diagnosis and for immunohistochemical staining. Histopathologic diagnosis of each neoplastic tissue was performed according to the World Health Organisation criteria by the Department of Pathology, Chiba University Hospital. Clinicopathologic staging was determined by the TNM classification of the International Union against Cancer. All patients had SCC or leukoplakia that was histologically confirmed, and tumour samples were checked to ensure that tumour tissue was present in more than 80% of the specimens.

### RNA isolation

Total RNA was extracted using Trizol Reagent (Invitrogen Life Technologies, Carlsbad, CA, USA) according to the manufacturer’s instructions. Each of extracted RNA was stored separately at −80°C until use.

### In-house cDNA microarray analysis

In total, 20 *μ*g of purified total RNA obtained from four randomly selected patients with TSCC was subjected to microarray analysis. Samples of total RNA from these patients were similarly purified and pooled to serve as controls. A cDNA microarray chip consisting of 2304 cDNAs was created as previously described (Moriya *et al*, 2003). The cDNA microarray was based on an oligo-capped cDNA library of the OSCC tissues, oral normal tissues and OSCC-derived cell lines: unique clones (2304) were selected from about 4608 sequenced clones in the library. cDNA microarray analysis was performed as described previously (Yoshikawa *et al*, 2000). Briefly, Cy3-dUTP or Cy5-dUTP (Amersham Biosciences UK Ltd, UK) was incorporated during reverse transcription of 20 *μ*g of purified total RNA, primed by an oligo dT primer. Different fluorescent-labelled probes from each tumour and normal tissues were mixed and applied to the cDNA microarray after incubation at 65°C overnight in a humidified atmosphere. Two hybridyzations were carried out for each experiments in which the fluorescent dyes were switched during cDNA synthesis. Each pair of corresponding probes was hybridysed to a separate microarray. The fluorescent images of hybridised microarrays were scanned with a fluorescence laser confocal slide scanner (Scan Array Lite, Packard Bio Science, Billerica, MA, USA). Images were analysed with microarray analysis software (QuantArray, Packard Bio Science), according to the manufacturer’s instructions. Spots were only included if their raw fluorescence intensities at least 1.5 times the local background.

### Real-time quantitative RT–PCR

qRT–PCR was used to examine the expression status of the 10 genes whose function is known.

The cDNA templates for qRT–PCR were synthesised from the four RNA samples of TSCCs used in the microarray analysis, and two additional RNA samples were prepared for the evaluation of *Rab1a* gene expression. The nucleotide sequences of gene-specific primers and predicted sizes of the resulting PCR products for qRT–PCR are shown in Table 1. qRT–PCR was performed with a single method using a LightCycler FastStart DNA Master SYBR Green I kit (Roche Diagnostics GmbH, Mannheim, Germany). For preparing the standard curve, 1.5 *μ*g of total RNA from normal oral tissue was reverse transcribed with Superscript™ reverse transcriptase (Life Technologies, Grand Island, NY, USA) and oligo-d(T)_12−18_ primer, after which serial dilutions were made corresponding to cDNA transcribed from 300, 30, 3.0, and 0.3 ng of total RNA. The PCR reactions using a LightCycler (Roche) apparatus were carried out in a final volume of 20 *μ*l of reaction mixture consisting of 2 *μ*l of FirstStart DNA Master SYBR Green I mix (Roche), 3 mM MgCl_2_, and 0.2 *μ*l of the primers, according to the manufacturer’s instructions. The reaction was performed as per the manufacturer’s instructions. The transcript amount for genes were estimated from the respective standard curves and normalised to the glyceraldehyde-3-phosphate dehydrogenase (GAPDH) transcript amount determined in corresponding samples.

### Immunohistochemistry

Out of the differentially upregulated genes, one gene (*Rab1a*) was singled out as a valid candidate biomarker of TSCC. In totl, 54 pairs of tongue carcinoma and matched normal tissue and 13 TLP paraffin-embedded tissue samples were used for immunohistochemistry to examine Rab1A protein expression status. Sections (4 *μ*m) were deparaffinised, unmasked Rab1A antigen by microwave treatment in 10 mM sodium citrate buffer (pH6) and rinsed three times in PBS solution. After quenching the endogenous peroxidase activity in 0.5% H_2_O_2_ for 30 min, the sections were reacted with Rab1A polyclonal antibody (Santa Cruz Biotechnology, Santa Cruz, CA, USA) at a dilution of 1 : 50 overnight at room temperature in a humidified atmosphere. Upon incubation with the primary antibody, the specimens were washed three times in phosphate-buffered saline and treated with ENVISION reagent (DAKO JAPAN Inc., Kyoto, Japan) followed by colour development in 3,3′-diaminobenzidine tetrahydrochloride (DAKO). Finally, the slides were lightly counterstained with haematoxylin, dehydrated with ethanol, cleaned with xylene, and mounted. As a negative control, duplicate sections were immunostained without exposure to primary antibodies. To quantitate the state of Rab1A protein expression, the mean percentage of positive tumour cells was determined in at least five random fields at × 400 magnification in each section. The intensity of the Rab1A-immunoreaction was scored as follows: 1+, weak; 2+, moderate; and 3+, intense. The percentage of positive tumour cells and the staining intensity then were multiplied to produce a Rab1A-immunohistochemical staining score. Cases with a Rab1A score >76.17 (the highest score of normal tissue) were defined as positive. These judgments were made by two independent pathologists, neither of whom had any knowledge or information pertaining to the patients’ clinical status. Any discrepancy in the scoring of the slides was resolved jointly by the pathologists by discussion and a consensus observation was recorded afterward. Statistical significance was evaluated by the Fisher’s exact test, or Mann–Whitney’s *U*-test.

## RESULTS

### Identification of candidate TSCC target genes

In the search for genes involved in the development of human TSCC, we compared gene expression between tumorous and normal tongue tissue samples in four patients with TSCC. The comparison between tumours and normal tissues showed that 16 genes were upregulated in all cases (Table 2).

### Validation of the in-house cDNA microarray results

**Figure 1:**
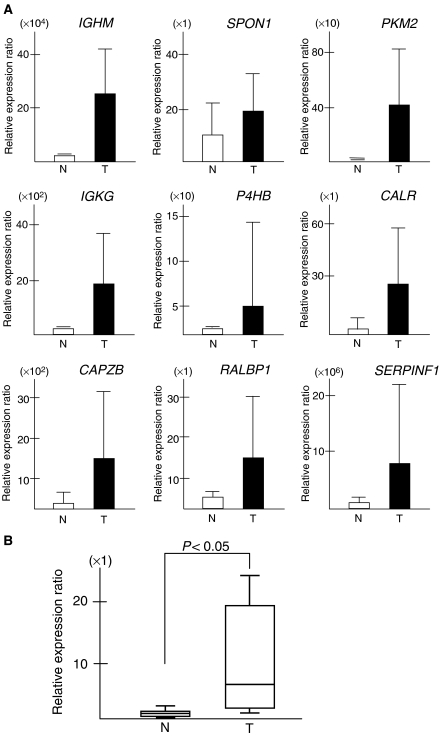
Validation of cDNA microarray data by real-time quantitative RT–PCR (qRT–PCR). (**A**) Nine genes with known molecular function were subjected to qRT–PCR in the mRNA from four TSCCs and four samples of the corresponding normal tissue used in the microarray analysis. A significant upregulation was evident in all the genes evaluated. (**B**) A significant higher expression of the *Rab1a* gene was detected in primary TSCCs (*n*=6) than that in the six corresponding normal tissues (*P*=0.0277, Wilcoxon signed-rank test). Relative expression ratio is defined as the expression levels of the gene to those of the internal reference gene, GAPDH. The assays were carried out in triplicate and means±standard deviations were plotted.

To further validate the cDNA array approach, we performed qRT–PCR on the 10 genes with known function (Table 1) to analyse mRNA expression level in the four TSCC cases examined by the cDNA microarray analysis. We found that all the 10 genes showed a significant upreguration in tumour tissues (Figure 1A, B) when compared to corresponding normal tissues, although the fold change in the expression level was not exactly same between the microarray analysis and qRT–PCR. In addition, there was a statistically significant difference of the *Rab1a* gene expression status between TSCCs (*n*=6) and matched normal tongue tissues (Wilcoxon signed-rank test, *P*=0.0277; Figure 1B). Futhermore, all the TSCCs showing upregulation of the *Rab1a* mRNA expression revealed overexpression of the protein.

### Expression of Rab1A in malignant and premalignant lesions of the tongue

**Figure 2:**
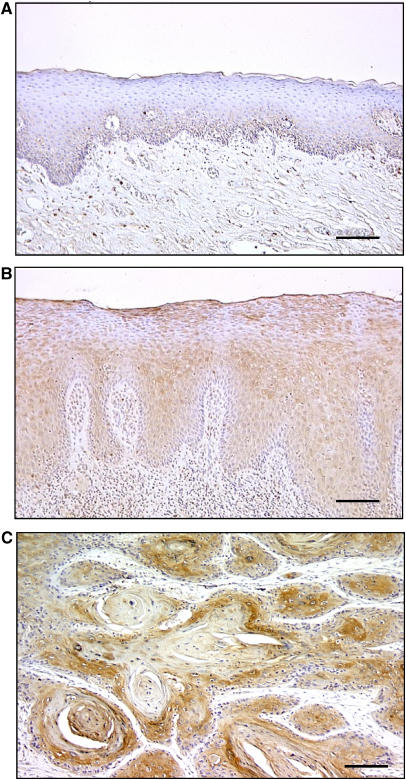
Immunohistochemical staining of Rab1A. (**A**) Normal tongue epithelium tissue shows weak expression of Rab1A protein. (**B**) Rab1A-positive case of TLP. The immunoreaction is slightly enhanced in the basal layer. (**C**) TSCC tissue shows strong cytoplasmic staining of the tumour cells. Original magnification, × 40. Bar=100 *μ*m.

**Figure 3:**
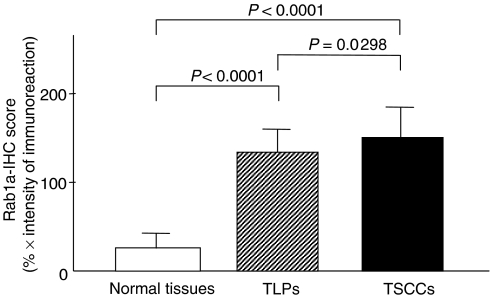
State of Rab1a protein expression in normal oral tissues (*n*=54), TLPs (*n*=13), and TSCCs (*n*=54). The Rab1A-IHC scores were calculated as follows: Rab1A-IHC=the mean percentage of positive tumour cells × staining intensity. Rab1A protein expressions in TLPs and TSCCs were significantly higher than in normal oral tissues (*P*<0.0001, Mann–Whitny’s *U*-test). There was also slight difference between TLPs and TSCCs (*P*=0.0268, Mann–Whitny’s *U*-test). Results are expressed as means±s.d.

In total, 54 patients with TSCC were identified for whom there was adequate histologic material available for immunohistochemical analysis. The correlation between the clinicopathologic characteristics of patients with TSCC and Rab1A expression status is summarised in Table 3. All normal oral mucosa specimens had no or significant downregulation of Rab1A expression and were considered as Rab1A-negative (Fisher’s exact test). Among the tumours examined, 53 of 54 cases (98%) had a Rab1A-immunoreaction in the cytoplasm of the tumour cells (Table 3). However, there was no statistically significant differences between Rab1A expression and the clinicopathologic features (Table 3). Interestingly, 12 of 13 TLPs (93%) were considered Rab1A-positive. Representative results for Rab1A protein expression in normal oral tissue, TLP, and primary TSCC are shown in Figure 2. The Rab1A immunohistochemistry scores for normal tissues, TLPs, and TSCCs ranged from 0 to 76.2 (mean, 31.9), 73.5 to 182.9 (mean, 121.4), and 60.2 to 221.8 (mean, 148.0), respectively. The Rab1A expression levels in primary TSCCs and TLPs were significantly higher than those in normal oral tissues (Mann–Whitney’s *U*-test, *P*<0.0001; Figure 3). There was also a significant difference in RAB1A-IHC scores between TSCCs and TLPs (Mann–Whitney’s *U*-test, *P*=0.0298; Figure 3).

## DISCUSSION

Considerable evidence has been reported that the identification of novel disease relevant targets/pathways and tumour classification/stratification can be achieved by microarray-based gene expression profiling. In this context, there are numerous studies of the use of disease profiles in tumours of clinically relevant subgroups, including the brain (Sallinen *et al*, 2000), oesophagus (Lu *et al*, 2001), lung (Wang *et al*, 2000), gastrointestinal tract (El-Rifai *et al*, 2001; Inoue *et al*, 2002), thyroid (Wasenius *et al*, 2003), breast (Assersohn *et al*, 2002; Ellis *et al*, 2002), leukaemia (Moos *et al*, 2002), colon (Williams *et al*, 2003), prostate (Bull *et al*, 2001). There are also several studies to classify OSCCs using commercially available cDNA microarrays (Ohyama *et al*, 2000; Alevizos *et al*, 2001; Kuo *et al*, 2002; Vigneswaran *et al*, 2005). We recently developed an in-house cDNA microarray derived from our oligo-capped human cDNA library prepared from noncancerous and cancerous tongue tissues, and oral cancer cell lines (Moriya *et al*, 2003).

The first step in the present study was to identify gene(s) that were differentially expressed in TSCC compared with normal tongue epithelium using the in-house cDNA microarray system. Defining a 2.0-fold difference as the threshold, 16 genes of interest were identified from four TSCC specimens. As shown in Table 2, 13 were known genes and three were unknown genes. In total, 10 genes (*IGHM*, *SPON1*, *PKM2*, *IGKG*, *P4HB*, *CALR*, *CAPZB*, *RALBP1*, *SERPINF1*, and *Rab1a*) with known molecular function were subjected to qRT–PCR analysis, and they were confirmed to be upregulated in the TSCCs examined, when compared to the corresponding normal tissues. So far, none of these genes or their corresponding proteins have been attributed directly to the development of TSCC, suggesting that these genes are novel potential targets for this disease.

*Immunoglobulin heavy constant mu* (*IGHM*) encodes the heavy chain unique to IgM that is on chromosome 14q32.33. Chromosome rearrangements of this band affect the *AKT1* gene, the proto-oncogene of the viral oncogene *v-akt* (Staal *et al*, 1988).

Spondin 1 (SPON1) is an extracellular matrix protein. Nagase screened human brain cDNAs for the potential to encode proteins that are at least 50 kDa, which they called KIAA0762 (Nagase *et al*, 1998). It is similar to rat F-spondin; thus, this gene may have a role in the growth and guidance of axons. They also indicated that the *SPON1* gene is on chromosome 11p15.2. Nearby are the *MUC2* and *MUC6* genes (11p15) that might be related to lymph node metastasis (Nishiumi *et al*, 2003).

Pyruvate kinase muscle type 2 (PKM2) is known as ATP:pyruvate phosphotransferase and occurs in four isozymic forms (L, R, M1, M2). Tsutsumi *et al* (1988) isolated and sequenced two overlapping clones covering the entire coding sequence of *PKM2*. Kress *et al* (1998) indicated that mRNA level of *PKM2* is increased in human colorectal cancers in comparison to the corresponding normal tissues.

*IGKG* encode immunoglobulin kappa chain constant region. Lenormand *et al* (1991) reported 20 of the 25 patients with B-cell chronic lymphocytic leukaemia (B-CELL) showed *IGKC* rearrangement.

P4HB is involved in hydroxylation of prolyl residues in preprocollagen. Tasanen *et al* (1988) isolated genomic clones for the human gene coding for this multifunctional protein. Pajunen *et al* (1987), 1988) assigned the gene to chromosome 17, specifically, 17q23–q25. The chromosomal aberration of this region may be involved in carcinogenesis in the tylosis with oesophageal cancer (TOC) (Shahabi *et al*, 2004) and liver cancer (Midorikawa *et al*, 2004).

CALR is a multifunctional protein that acts as a major Ca(2+)-binding (storage) protein in the lumen of the endoplasmic reticulum. Accumulation of CALR protein is observed in a few cancers, including breast cancer (Franzen *et al*, 1996) and hepatocellular carcinoma (Yoon *et al*, 2000).

Capping protein muscle Z-line, beta (CAPZB) is a member of the F-actin capping protein family. Barron-Casella *et al* (1995) isolated cDNAs homologues for the beta subunit of chicken *Cap* Z from human retinal cDNA libraries. This gene encodes the beta subunit of the barbed-end actin binding protein that regulates growth of the actin filament by capping the barbed end of growing actin filaments. Those investigators mapped the *CAPZB* gene to 1p36.1, which has frequent loss of heterozygosity observed in neuroblastomas (Fong *et al*, 1989) and in oropharyngeal epithelial carcinomas (Grati *et al*, 2000).

Jullien-Flores *et al* (1995) obtained a cDNA encoding *RALBP1*, which they termed *RLIP76*. *RALBP1* participate in signalling for a variety of cellular processes and are regulated in part by guanine nucleotide dissociation stimulators, and coordinate the cellular actions of activated EGF receptors and Ral-GTPases. The activity of *RALBP1* may contribute to the drug-resistant of small-cell lung cancer (SCLC) (Singhal *et al*, 2003).

Serpins are a group of serine protease inhibitors, some of which have also been reported to exhibit neurotrophic activity. In studies aimed at identifying antiangiogenic factors in the eye, Dawson *et al* (1999) identified SERPINF1. SERPINF1 may serve as a multifunctional antitumour agent in neuroblastomas, inhibiting angiogenesis (Crawford *et al*, 2001).

*Rab1a* is a member of the *Ras* oncogene superfamily. Rab proteins represent a family of at least 30 different Ras-like GTPases that function in the processes by which membrane vesicles identify and/or fuse with their targets (Zahraoui *et al*, 1989; Ferro-Novick and Novick, 1993; Novick and Brennwald, 1993; Jaakko *et al*, 1995), suggesting that abnormal regulation of membrane traffic, which is one of the important cellular processes, may lead to tumorigenesis. Thus, we have selected the *Rab1a* gene for further investigation. To clarify its relative contribution to tongue carcinogenesis, we further investigated the protein expression in a series of TSCCs and TLPs. We detected a comparatively strong tumour cell-localised cytoplasmic Rab1A-immunoreaction, raising the possibility that the gene product(s) may serve as a diagnostic marker of tongue cancer. By evaluating the Rab1A immunohistochemistry scores using the Mann–Whitney’s *U*-test, significant Rab1A upregulation was evident not only in the primary TSCCs (*P*<0.0001, TSCC *vs* corresponding normal tissues) but also in the TLPs (*P*<0.0001, TLPs *vs* corresponding normal tissues) when compared with normal tissues. In addition, the levels of Rab1A expression in SCCs tended to be higher than that in TLPs (*P*=0.0268), suggesting that Rab1A expression is not only a precipitous event during tongue carcinogenesis but also an important candidate for the progression of TSCCs. In addition, whereas most corresponding normal tongue squamous cells appeared not to express Rab1A, faint but positive cytoplasmic staining was detected in a basal layer of tongue epithelial cells of matched normal tongue tissue (Figure 2A). One possible explanation is that Rab1A could be associated with proliferation state (even of normal epithelium). Thus, it might be a secondary event not directly associated with cancer causes. At present, it is unclear why basal layer of normal epithelium showed Rab1A protein stimulation. Further studies on a large series of patients and in samples of a proliferative, noncancererous disorder, will provide more accurate information about the involvement of Rab1A expression in the development of TSCC.

Overall, we found expression profile changes that suggest genetic alterations not only favouring a transformed epithelial cell *per se* but also having the capabilities of modulating the immediate microenvironment, which may aid tumour progression in the tongue. The present study identified aberrant Rab1A expression in premalignant and malignant tissues, suggesting its appearance and overexpression in TLPs as a possible biologic marker of imminent progression. The association of genes identified in our study with clinical variables and gaining an understanding of the regulation of their expression will aid in determining their potential use as molecular markers in this cancer.

## Figures and Tables

**Table 1:** Primer pairs for quantitative RT–PCR analysis

**Gene^a^**	**Forward primer**	**Reverse primer**
IGHM	5′-TCAGTAGACAGCTCCAAGACCAG-3′	5′-CCCAGACGTCCATACCGTAGTAGTAGT-3′
SPON1	5′-CTCTTCCTGCAGAGGAGTAGTGTCA-3′	5′-CTGGGACTCAGGCATAGTCACTTC-3′
PKM2	5′-GGAATGAATGTGGCTCGTCTG-3′	5′-CCAGCGTGATTTTGAGAGTGG-T-3′
IGKC	5-AGCCTTGATCCTTGGGAATC-3′	5′-GAAAGCCCTGAGGCTTCTCT-3′
P4HB	5-ACAGCTTCCCCACACTCAG-3′	5′-GGGTCTGGCTTTGCGTATTA-3′
CALR	5-GTTTCGAGCCTTTCAGCAC-3′	5′-GAAGTCCCAATCGTCTTCCA-3′
CAPZB	5′-TGGAGGTGGAAGCCAACAAT-3′	5′-CGCTGGATTTCTCCTGCACT-3′
Rabla	5′-TATGGGACACAGCAGGCCAGG-3′	5′-ACGGAATTCCAAGGGAATCAGC-3′
RALBP1	5′-CTGGTGGACTCCCAATTGAC-3′	5′-GTGTGGGGTTTGTTTTCGAC-3′
SERPINF	5′-GCTGTCTCCAACTTCGGCTA-3′	5′-GTAGAGAGCCCGGTGAATGA-3′
GAPDH	5′-CATCTCTGCCCCCTCTGCTGA-3′	5′-GGATGACCTTGCCCACAGCCT-3′

a IGHM=immunoglobulin heavy constant mu; SPON1=spondin 1; PKM2=pyruvate kinase muscle type 2; IGKC=immunoglobulin kappa constant region; P4HB=procollagen-proline,2-oxoglutarate-4-dioxygenase,beta; CALR=calreticulin; CAPZB=capping protein muscle Z-line, beta; RALBP1=rala-binding protein 1; SERPINF=pigment epithelium-derived factor.

**Table 2:** Genes with high expression levels in TSCCs

**UniGene ID**	**Gene**	**Molecular function**	**Chromosomal position**	**Fold change^a^**
Hs.297962	Hypothetical protein	Unknown	Chromosomes 19	10.79
Hs.153261	1GHM	Immune system	14q32.33	9.02
Hs.5387	SPON1	Extracellular matrix protein	Ilpl4–pl5.2	5.92
Hs.198281	PKM2	Catalyse the production of phosphoenolpyruvate	15q22	5.66
Hs.406565	1GKC	Immune system	2pl2	5.25
Hs.90315	KIAA0007	Unknown	2p23.2	4.61
Hs.55098	C3orf6	Unknown	Chromosomes 3	3.24
Hs.43431	Hypothetical protein	Unknown	6p22.3	2.92
gb∣BX442293^b^	Unknown	Unknown	Unknown	2.82
Hs.83286	Hypothetical protein MGC33424	Unknown	Unknown	2.61
Hs.410578	P4HB	Protein disulphide isomerase	17q25	2.57
Hs.353170	CALR	Calciumstrage, transcriptin co-repressor	19pl3.3–pl3.2	2.57
Hs.333417	CAPZB	Actin binding	lpG6.1	2.45
Hs.227327	Rabla(RAS oncogene family)	RAB small monomeric GTP ase	2pl4	2.27
Hs.75447	RALBP1	GTPase activator	Ispll.3	2.22
Hs.173594	3ERP1NF1	Serine protease inhibitor	17pl3.1	2.16

a Fold over-expression for rnicroarray data based on ratio of fluorescence for TSCCs compared to normal control.

b GenBank Accession Number.

**Table 3:** Correlation between Rabla expression and clinicopathologic features in human oral cancer

**Clinical classification**	**Total**	**Result of immunostaining (no. of patients, %)**		
		**RablA(+)**	**RablA(−)**	***P*-value**
*Age at surgery*				
<60	27	27 (100)	0 (0)	0.24074
60–70	14	14 (100)	0 (0)	
⩾70	13	12 (92)	1 (8)	

*Gender*				
Male	35	34 (97)	1 (3)	1
Female	19	19 (100)	0 (0)	

*T of primary tumor*				
Tl	6	6 (100)	0 (0)	0.33333
T2	18	18 (100)	0 (0)	
T3	18	18 (100)	0 (0)	
T4	12	11 (92)	1 (8)	

*N of regional lymph node*				
K (+)	40	39 (98)	1 (2)	1
K (−)	14	14 (100)	0 (0)	

*Stage*				
I	5	5 (100)	0 (0)	1
II	5	5 (100)	0 (0)	
III	16	16 (100)	0 (0)	
IV	28	27 (96)	1 (4)	

*Histopathological type*				
Well differentiated	35	34 (97)	1 (3)	1
Moderately differentiated	9	9 (100)	0 (0)	
Poorly differentiated	10	10 (100)	0 (0)	
Leukoplakias	13	12 (92)	1 (8)	
